# Acquired Hemophilia Secondary to Soft-tissue Sarcoma: Case Report from a Latin American Hospital and Literature Review

**DOI:** 10.7759/cureus.2621

**Published:** 2018-05-14

**Authors:** Camila Casadiego-Peña, Alejandro González-Motta, Oliver G Perilla, Pedro D Gomez, Leonardo J Enciso

**Affiliations:** 1 Department of Radiation Oncology, Instituto Nacional de Cancerologia, Universidad Militar Nueva Granada, Bogota, COL; 2 Department of Hematology, Instituto Nacional De Cancerologia, Bogota, COL; 3 Faculty of Medicine, Universidad Nacional De Colombia, Bogota, COL; 4 Department of Internal Medicine, Instituto Nacional De Cancerologia, Bogota, COL

**Keywords:** acquired hemophilia, secondary hemophilia, soft tissue sarcoma

## Abstract

Acquired hemophilia A is a rare bleeding disorder caused by inhibiting antibodies against factor VIII characterized by the presence of severe bleeding, which in occasions can be lethal. The bleeding manifestations typically have a sudden onset and patients have a negative family and personal histories of bleeding, with a normal prothrombin time (PT) and an extended partial thromboplastin time (PTT). Incidence has been calculated to be between 0.2 and 1.48 cases per million per year. Between 6% and 15% of cases are associated with neoplasms. Here, we present a 52-year-old male with back myxofibrosarcoma who developed acquired hemophilia without response to treatment used and ultimately died. The most common cancers associated with acquired hemophilia are lung and prostate cancer. We found one other case of a patient with Kaposi's sarcoma that was unassociated with HIV infection who presented with severe postoperative bleeding. For bleeding in acquired hemophilia A, the treatments of choice are “bypass” agents, such as recombinant-activated factor VIII (rFVIIa) or activated prothrombin complex concentrate. Any delay in the start of treatment or the usage of insufficient doses is associated with the progression of bleeding symptoms and worsening general condition. In the case of acquired hemophilia secondary to neoplasia, it is recommended that immunosuppressive therapy to eradicate the inhibitors be combined with treatment for the underlying neoplastic disease. In our patient, it was not possible to offer a surgical treatment that enabled the control of the neoplasia, nor he was considered a candidate for chemotherapy or radiotherapy, limiting the treatment to immunosuppressive and “bypass” management.

## Introduction

Acquired hemophilia A is a rare bleeding disorder characterized by the presence of inhibiting autoantibodies against coagulation factor VIII [1]. Between 10% and 15% of cases are associated with neoplasms, which can be diagnosed before, during, or after bleeding manifestations [2-3]. A fast and accurate diagnosis and the prompt beginning of treatment are required, preferably with bypass agents to control bleeding that is often life-threatening [4]. Additionally, inhibitor eradication therapy must be initiated, as well as treating the underlying cause [4]. The case of a patient with acquired hemophilia associated with the presence of myxofibrosarcoma is presented, and a literature review is performed.

## Case presentation

A male, 52-year-old patient visited the Instituto Nacional de Cancerología (INC) in Bogotá, Colombia, because of the development of a mass in the back region. There had been progressive growth and multiple drainage attempts due to a suspected abscess at various referral sites without relief. He was given non-oncological surgical resection in another institution, and his pathology report was compatible with high-grade sarcoma with positive margins. Additionally, he referred to bilateral vision loss that had been evolving for one month. The initial clinical exam revealed a patient with normal vital signs who was disoriented with regard to space and time though awake and had poor communication with the examiner. He presented a hyperpigmented tumor measuring 10 x 8 cm in the back region, along with a scarred postsurgical lesion without inflammatory signs. The biopsy was reviewed by the INC Pathology Department, which indicated a high-grade sarcoma compatible with myxofibrosarcoma.

**Figure 1:**
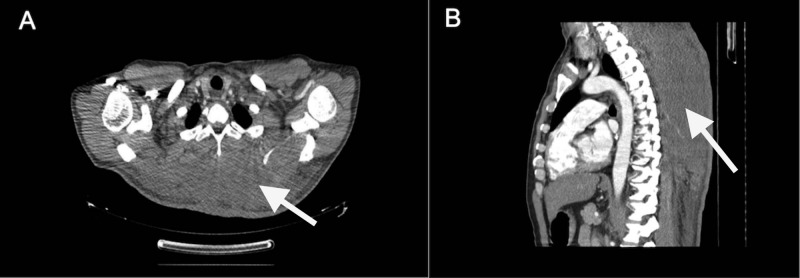
Chest CT Scan A chest CT scan with contrast.  White arrows showed an undefined heterogeneous mass dependent on the soft tissues in the posterior thorax wall, with infiltration signs in the muscular plane and the density alteration of the subcutaneous cellular tissue and skin. A) Axial view. B) Sagittal view.

He was examined by the Ophthalmology Department, which considered bilateral retinal detachment probable. An abdominal computed tomography (CT) scan was performed, which showed a diffuse density alteration of the subcutaneous cellular tissue, without evidence of lesions, probably due to edema. A chest computed tomography (CT) scan with contrast showed an undefined heterogeneous mass dependent on the soft tissues in the posterior thorax wall, with infiltration signs in the muscular plane and the density alteration of the subcutaneous cellular tissue and skin (See Figure 1). He was examined by clinical oncology, surgical oncology, and radiation oncology services, which indicated he was not a candidate for chemotherapy, surgical treatment, or radiotherapy because of the significant extension of the tumor into the upper and lower back, the inadequate response of these tumors to chemotherapy, and the patient’s poor Zubrod performance score.

During inpatient care, the patient showed substantial clinical worsening characterized by the progressive deterioration of his consciousness state and persistent oral cavity bleeding. Laboratory tests were requested, which showed the prolongation of blood clotting time due to extended partial thromboplastin time (PTT), with the test value for prothrombin time (PT) being 11.9 seconds and that for PTT being 50.1 seconds (normal value until 28 seconds). The mixing study showed PTT fixing with a 50:50 normal plasma sample that lasted for the first and second hour, suggesting intrinsic pathway coagulation factor deficiency. Even though the mixing study was not suggestive of the presence of inhibitors, the medical history of the patient, the presence of an active neoplasm, and the recent beginning of bleeding created a clinical suspicion of acquired hemophilia. For these reasons, a new mixing study, VIII factor assay, and Bethesda assay to measure factor VIII inhibitors were requested. In addition, treatment with prednisone at 1 mg/kg/day and factor VIII at 1700 international unit (IU) every 12 hours was initiated. The results of these assays showed a basal PTT of 67 seconds, which with the 50:50 sample with normal plasma, was reduced to 38,2 seconds and maintained at around normal values two hours afterwards, with a value of 36,3 seconds. Factor VIII was measured at 4% and remained at this same value (4%) 10 minutes after the injection of intravenous factor VIII. The specific factor VIII inhibitor assay reported 1.6 Bethesda units. A diagnosis of acquired hemophilia was made based on the presence of hidden low-titer inhibitors in the mixing study.

Based on the findings above, the persistence of oral cavity bleeding, and daily transfusion support in the form of two red blood cell units, IV treatment with an activated prothrombin complex like the factor eight (VIII) inhibitor bypassing activity (FEIBA) at a dose of 3500 units/12 h (50 U/kg every 12 hours) was initiated. During the patient’s evolution, hypofibrinogenemia development, the continuous elevation of D-dimer levels, and progressive thrombocytopenia suggested a diagnosis of disseminated intravascular coagulation (DIC). Because of the unresolved bleeding, it was decided to initiate a transfusion of cryoprecipitate and tranexamic acid oral rinse while augmenting the FEIBA dose. The patient developed a low response to the established treatment, along with more profound neurological impairment (central nervous system bleeding was ruled out) and the persistence of red blood cell, platelet, and cryoprecipitate transfusion requirements. Additionally, the patient had severe hyponatremia in the form of an electrolyte imbalance. Electrolyte correction was initiated, and because of the persistence of bleeding, oral cyclophosphamide was added to the immunosuppressive treatment, with the objective of eradicating the inhibitors. Seven days afterward, because of the lack of clinical response to management and the impossibility of providing specific antineoplastic treatment, with the agreement of the patient´s family, transfusion support was suspended, and palliative care was initiated. The patient died one week after.

## Discussion

Acquired hemophilia A is a rare autoimmune disease caused by inhibiting antibodies against factor VIII, usually the immunoglobulin G1 (IgG1) and the IgG4 subclasses [1]. The disease is characterized by the presence of severe and unexpected bleeding, which can be life-threatening [1]. Inhibiting antibodies against every coagulation factor have been reported, with factor VIII inhibitors being the most frequent [5]. The bleeding manifestations typically have a sudden onset, and generally, patients have negative family and personal histories of bleeding, along with a normal PT and extended PTT [6]. Incidence has been calculated as between 0.2 and 1.48 cases per million per year. These cases follow a biphasic distribution, with an initial peak between 20 and 30 years (this peak has female predominance associated with pregnancy and collagen vascular disorders) and a larger second peak in those older than 60 years (both men and women which is related with a higher incidence of underlying neoplasia) [1].

The European Acquired Hemophilia Registry (EACH2) is the largest reported cohort of patients with acquired hemophilia A [7-8]. This report included 501 patients (53% male, 47% female) who were diagnosed between 2003 and 2008 in 117 health centers in 13 European countries. Their median age was 74 years. Approximately half of the patients had an underlying disease: malignancies (12%), autoimmune diseases (12%), pregnancy (8%), infections (4%), drugs (3%), and other disorders (15%).

In other reports, association with cancer was found in 14.6% of patients [2]. Chronic lymphocytic leukemia represents 30% of the hematological malignancies associated with acquired hemophilia A, while the prostatic and lung carcinoma, represent each of them, 25% of the solid tumors associated with this disease [3]. The most frequent circumstance is that the neoplasia is diagnosed during or before the hemophilia is diagnosed [3]. In infrequent cases, the diagnosis of hemophilia precedes the diagnosis of neoplasia [3]. Cancer presence worsens the prognosis compared to patients without neoplasia. A meta-analysis from 2009, showed an increase of all causes of mortality with an OR of 2.76 (CI 1.38–5.5) [9]. A recent systematic review [10] found that among 105 patients with cancer and acquired hemophilia, the mean age was 68 years old. Skin and mucosal bleeding were the most prevalent symptoms. Of the solid tumors, prostate cancer was the commonest cancer diagnosis, followed by lung cancer. The most common hematologic cancers were lymphoma, chronic lymphocytic leukemia, and plasma cell dyscrasias [10]. In the current report, we show a patient with soft tissue sarcoma associated with acquired hemophilia. In the literature review, we found only one case of a patient with Kaposi´s sarcoma unassociated with human immunodeficiency virus (HIV) infection, which presented as severe postoperative bleeding [11].

In the EACH [7-8] the 95% of patients with acquired hemophilia A of EACH2 debuted with a bleeding event, usually spontaneous hemorrhages (77%), trauma-associated bleeding (8%), surgery (8%) or labor (4%). The most frequent site of bleeding was skin (53%) followed by muscular bleeding (50%) and mucous tissue bleeding (32%). Joint (5%) and central nervous system (1%) bleeding were infrequent. In 70% of cases the bleeding was classified as severe (defined as a hemoglobin level below 8 g/dL, a fall in hemoglobin level >2 g/dL, muscular, retroperitoneal or intracranial bleeding, or that compromises limb viability). The median time between the bleeding event until the diagnosis was three days. Patients with more severe bleeding had higher inhibitor titers, lower hemoglobin levels and deeper tissue bleeding events [8].

Differential diagnoses include other causes of extended PTT, such as treatment with heparin or dabigatran, as well as deficiencies in or acquired inhibitors of various components of the intrinsic pathway, such as factors IX or XI. Other causes of extended PTT include lupus anticoagulant, as well as deficiency in or inhibitors of factor XII, prekallikrein, or high-molecular-weight kininogen, which are usually not associated with bleeding. In contrast, lupus anticoagulant can be associated with bleeding, especially in the presence of antibodies against prothrombin. Heparinoids are also associated with bleeding and are characterized by extended PT and PTT [6]. For diagnosis, the following should be taken into account: First, measurement of diminished levels of factor VIII activity; second, normal PTT based on a mixing study (mixing the patient’s plasma at a 1:1 ratio with normal plasma). Because the antibodies involved in acquired hemophilia A are temperature- and time-dependent, the PTT results should be verified after one or two hours of incubation. Typically, extended PTT is detected in the mixing study after the incubation period and not immediately, as in the case of other inhibitors; finally, confirming the determination of the inhibitor using a Bethesda assay, the Nijmegen modification, or an enzyme-linked immunosorbent assay (ELISA) methodology. Our case was atypical given the mixing study was suggestive of factor deficiency. However, the clinical presentation was very suggestive of acquired inhibitors of factor VIII because the requested Bethesda assay was being positive.

The treatment of acquired hemophilia has two objectives: treating or preventing active bleeding and initiating the eradication of the inhibitor as soon as possible [12-13]. In the context of acquired hemophilia associated with cancer, the concurrent treatment of the underlying malignancy should be promptly initiated when possible [10]. Procedures that could cause iatrogenic bleeding should be avoided. In our patient, it was not possible to offer a surgical treatment that enabled the control of the neoplasia, nor he was considered as a candidate to chemotherapy or radiotherapy, limiting the treatment options.

In the treatment of bleeding, the following should be taken into account:

- Despite reports showing some percentage of patients treated with factor VIII, this is not effective, especially for the treatment of severe bleeding. The same is true for desmopressin [13].

- The treatments of choice are the “bypass” agents, such as recombinant-activated factor VII (rFVIIa) or activated prothrombin complex concentrate. Any of these can be used as first-line therapies, and an alternative product can be used if there is no response to the first agent. There are no head-to-head prospective comparative studies regarding these two therapies. The rFVIIa dose is 90 μg/kg every two–three hours, which should be titrated according to response, extending the dosing intervals to every four, six, eight, or twelve hours after controlling the bleeding. The applied dose of activated prothrombin complex concentrate is 50–100 U/kg every 12 hours. An effectivity above 90% is expected, according to the EACH2 report [4].

- Both agents are well-tolerated, with a thrombotic event rate of about 4.8% and a mortality rate from bleeding of 3.3%, according to data extracted from the EACH2 [7-8].

- Tranexamic acid can be used as adjunctive therapy to bypass agents, but the fact that this can augment thrombosis risk can be taken into account [14].

- A delay in the start of treatment or the usage of insufficient doses is associated with the progression of bleeding symptoms and worsening general condition [12].

The EACH2 [7] reported that complete remission (defined as a complete lack of bleeding, factor VIII activity levels above 50% after suspending all hemostatic agents for 24 hours, inhibitor non-detection, and steroid dosage below 15 mg/day) was more likely for patients who received steroids and cyclophosphamide (80%) as compared to monotherapy with steroids (58%) or rituximab-based therapies (62%). No differences in overall survival rate were found between the distinct management approaches. The rate of adverse effects was significantly higher for treatment with steroids and cyclophosphamide (41% vs 25%), mostly due to higher rates of infections and neutropenia [7]. Adverse thrombotic events secondary to factor VIII inhibitor bypass activity (FEIBA) were reported in the 4.8% and the 11.7% of patients in the EACH2 [4] and FEIBAC [15], respectively. Thrombosis secondary to recombinant factor VIIa use was found in 2.9% of patients in the EACH2 registry [4].

According to two international consensuses [12-13], prednisone should be initiated at 1 mg/kg/day for weeks one–three to eradicate the inhibitors. If at least a partial response is not achieved (defined as the absence of bleeding and factor VIII activity above 50% unrelated with hemostatic agents), treatment should be escalated to steroid/cyclophosphamide at 1.2 mg/kg/day for three more weeks and, if there is still no response, steroid/rituximab for weeks 7–10. In case of relapse, after achieving complete or partial response, prednisone must be reinitiated at the last effective dose, and according to the patient’s response, immunosuppressive therapy should be reinstated. The median response time is approximately five weeks [12-13]. Rituximab may be considered if prednisone and cyclophosphamide are contraindicated or not effective [12-13].

In the literature, there are case reports that describe the use of mycophenolate mofetil, azathioprine, vincristine, cyclosporine, plasmapheresis with or without extracorporeal immunoadsorption [16]. Immune tolerance induction protocols with high-dose factor VIII have been used as alternative treatments with successful results [16-17]. However, the advantage over other treatment options is unclear [18]. A recent phase II/III clinical trial demonstrated the efficacy of B domain deleted recombinant porcine factor VIII in the treatment of severe bleeding episodes in acquired hemophilia [19]. However, only a small number of subjects have been treated due to high price and difficulties involved in administration logistics and treatment follow-up [10]. High factor VIII activity levels and low-titer inhibitors (less than 16 BU/mL) have been associated with shorter time periods to achieve remission and better overall survival rates [20]. Spontaneous remission occurs in about one-third of cases and is usually related to postpartum, drug-induced inhibitors and patients with low titer inhibitors [1]. Acquired hemophilia A that occurs during the peripartum period has the best prognosis given its higher remission rates and better response to steroid treatment [1]. Once the patient achieves a complete response to the inhibitor eradication therapy, it is recommended to monitor the patient, requesting PTT and factor VIII levels monthly for the first six months; then at every two-three months for a year; and after that, at every six months. Relapse occurs in 15–24% of cases [1].

The mortality rate fluctuates between 31% in the EACH2 report [7] to 41% in the British report [2]. The leading cause of death is infection, especially infection associated with the use of immunosuppressive agents and steroids [2, 7]. However, in the first few weeks, hemorrhage is the most common cause of death, typically due to gastrointestinal, lung, intracranial, or retroperitoneal bleeding [1].

## Conclusions

In the context of a bleeding patient, acquired hemophilia associated with cancer is a crucial diagnosis to keep in mind. Unfortunately, our patient developed a terminal neoplastic disease, with a poor prognosis, and did not show a response to bypass agents and immunosuppressive therapies. In other patients, the prognosis of the disease may be better given the quick identification of inhibitors as the cause of bleeding, the early identification and treatment of neoplastic disease, and adequate hemostatic support and immunosuppressive therapy.

## References

[1] Webert KE (2012). Acquired hemophilia A. Semin Thromb Hemost.

[2] Collins PW, Hirsch S, Baglin TP (2007). Acquired hemophilia A in the United Kingdom: a 2-year national surveillance study by the United Kingdom Haemophilia Centre Doctors' Organisation. Blood.

[3] Reeves BN, Key NS (2012). Acquired hemophilia in malignancy. Thromb Res.

[4] Baudo F, Collins P, Huth-Kuhne A (2012). Management of bleeding in acquired hemophilia A: results from the European Acquired Haemophilia (EACH2) Registry. Blood.

[5] Kessler CM, Knobl P (2015). Acquired haemophilia: an overview for clinical practice. Eur J Haematol.

[6] Toschi V, Baudo F (2010). Diagnosis, laboratory aspects and management of acquired hemophilia A. Intern Emerg Med.

[7] Collins P, Baudo F, Knoebl P (2012). Immunosuppression for acquired hemophilia A: results from the European Acquired Haemophilia Registry (EACH2). Blood.

[8] Knoebl P, Marco P, Baudo F (2012). Demographic and clinical data in acquired hemophilia A: results from the European Acquired Haemophilia Registry (EACH2). J Thromb Haemost.

[9] Bitting RL, Bent S, Li Y, Kohlwes J (2009). The prognosis and treatment of acquired hemophilia: a systematic review and meta-analysis. Blood Coagul Fibrinolysis.

[10] Napolitano M, Siragusa S, Mancuso S, Kessler CM (2018). Acquired haemophilia in cancer: A systematic and critical literature review. Haemophilia.

[11] Cashin P, Lundberg LG, Hagberg H, Ejerblad E, Karlbom U (2010). Acquired haemophilia A and Kaposi's sarcoma in an HIV-negative, HHV-8-positive patient: a discussion of mechanism and aetiology. Acta Haematol.

[12] Collins P, Baudo F, Huth-Kuhne A (2010). Consensus recommendations for the diagnosis and treatment of acquired hemophilia A. BMC Res Notes.

[13] Huth-Kühne A, Baudo F, Collins P (2009). International recommendations on the diagnosis and treatment of patients with acquired hemophilia A. Haematologica.

[14] Tiede A, Scharf RE, Dobbelstein C, Werwitzke S (2015). Management of acquired haemophilia A [article in German]. Hamostaseologie.

[15] Borg JY, Negrier C, Durieu I (2015). FEIBA in the treatment of acquired haemophilia A: results from the prospective multicentre French 'FEIBA dans l'hemophilie A acquise' (FEIBHAC) registry. Haemophilia.

[16] Shetty S, Bhave M, Ghosh K (2011). Acquired hemophilia. Autoimmun Rev.

[17] Zeitler H, Ulrich-Merzenich G, Panek D (2012). Extracorporeal treatment for the acute und long-term outcome of patients with life-threatening acquired hemophilia. Transfus Med Hemother.

[18] Mulliez SM, Vantilborgh A, Devreese KM (2014). Acquired hemophilia: a case report and review of the literature. Int J Lab Hematol.

[19] Kruse-Jarres R, St-Louis J, Greist A (2015). Efficacy and safety of OBI-1, an antihaemophilic factor VIII (recombinant), porcine sequence, in subjects with acquired haemophilia A. Haemophilia.

[20] Tiede A, Klamroth R, Scharf RE (2015). Prognostic factors for remission of and survival in acquired hemophilia A (AHA): results from the GTH-AH 01/2010 study. Blood.

